# Supplementary material for: Exposure to live saprophytic Leptospira before challenge with a pathogenic serovar prevents severe leptospirosis and promotes kidney homeostasis
Source: eLife. 2024 Nov 11;13:RP96470. doi: 10.7554/eLife.96470 (PMC11554301; doi:10.7554/eLife.96470)
Supplement: Supplementary file 1. [file elife-96470-supp1.docx]

Supplementary File 1: Detailed list of primary fluorochrome conjugated antibodies used in flow cytometry.

| **Marker** | **Fluorophore** | **Company** |
| --- | --- | --- |
| CD45 | Brilliant Violet 605 | Biolegend |
| CD3 | Violet Fluor 450 | TONBO biosciences |
| CD19 | Brilliant Violet 785 | Biolegend |
| CD49b | PE Dazzle 594 | Biolegend |
| CD4 | PE | Biolegend |
| CD8 | APC-Cy7 | Biolegend |
| CD44 | APC | Biolegend |
| CD62L | PE-Cy7 | Biolegend |
